# Statement of retraction: Ang II enhances tubular cell Ets-1 expression and associated down stream signaling is mediated through AT1 receptors

**DOI:** 10.1080/0886022X.2026.2641849

**Published:** 2026-03-17

We, the Editors and Publisher of Renal Failure, have retracted the following article:

Kumar, D., Luan, L., Pathak, S., Salhan, D., Magoon, S., & Singhal, P. C. (2010). Ang II enhances tubular cell Ets-1 expression and associated down stream signaling is mediated through AT1 receptors. *Renal Failure, 32*(8), 986–991. https://doi.org/10.3109/0886022X.2010.501936

Since publication, concerns were identified by a third-party regarding the integrity of Figure 2. Further investigation by the publisher and journal has confirmed the integrity concerns. When approached for an explanation, due to the time elapsed since the study was conducted and institutional data retention policies, the authors stated they were unable to provide the raw data.

As verifying the validity of published work is core to the integrity of the scholarly record, we are therefore retracting the article. The corresponding author has been informed.

We have been informed in our decision-making by our editorial policies and the COPE guidelines.

The retracted article will remain online to maintain the scholarly record, but it will be digitally watermarked on each page as ‘Retracted’.

